# Gut Microbial Metabotypes Shape Polyphenol Bioactivity: Toward Precision Nutrition Strategies for Human Health

**DOI:** 10.3390/nu18142396

**Published:** 2026-07-22

**Authors:** Pasquale Perrone, Stefania D’Angelo

**Affiliations:** 1 Department of Psychology and Health Sciences, Pegaso Telematic University, 80143 Naples, Italy; pasquale.perrone@unipegaso.it; 2 Department of Medical, Human Movement, and Well-Being Sciences (DiSMMeB), Parthenope University of Naples, 80133 Naples, Italy

**Keywords:** gut microbiota, polyphenols, microbial metabotypes, bioavailability, bioactive metabolites, precision nutrition, mediterranean diet, metabolic health, urolithins, interindividual variability

## Abstract

The biological effects of dietary polyphenols are increasingly understood to depend not only on their intake, but also on their bioavailability, host metabolism, and gut microbiota-mediated biotransformation. Because many native polyphenols are poorly absorbed in the upper gastrointestinal tract, a substantial fraction reaches the colon, where intestinal microorganisms convert them into lower-molecular-weight metabolites, including urolithins, phenyl-γ-valerolactones, phenolic acids, enterolignans, and equol. These metabolites may display distinct absorption profiles and biological activities compared with their parent compounds, although their physiological relevance differs according to metabolite class, exposure level, and supporting evidence. Interindividual differences in gut microbiota composition and function contribute to substantial variability in metabolite production, leading to the concept of microbial metabotypes, which classify individuals according to their capacity to generate specific microbial-derived metabolites. This narrative review critically examines gut microbiota-mediated polyphenol biotransformation, major microbial metabotypes, determinants of metabotype variability, and their potential relevance for precision nutrition. Particular attention is given to urolithin, equol, and enterolignan metabotypes while distinguishing relatively well-characterized models from emerging or insufficiently standardized metabolic phenotypes. We also discuss the level of evidence supporting biological effects related to mitochondrial function, endothelial homeostasis, metabolic regulation, inflammation, gut barrier integrity, and gut–brain communication, distinguishing human evidence from preclinical and mechanistic findings. Although metabotype-guided approaches may improve responder stratification in future nutritional studies, their clinical translation remains limited by heterogeneous challenge protocols, non-standardized analytical cut-offs, incomplete validation across populations, and insufficient long-term intervention data. Therefore, microbial metabotypes should currently be considered as promising functional biomarkers for research and stratified trial design rather than established tools for routine personalized dietary prescription.

## 1. Introduction

Precision nutrition has emerged from the recognition that individuals do not respond uniformly to the same dietary patterns, foods, or bioactive compounds. Conventional nutritional recommendations are generally based on population-level evidence and often assume relatively homogeneous responses among individuals. However, increasing evidence indicates that metabolic, vascular, inflammatory, and neurocognitive responses to dietary interventions may vary substantially between subjects, even when exposed to similar nutritional stimuli [1,2]. This interindividual variability represents a major limitation of the traditional “one-size-fits-all” nutritional model and highlights the need to identify biological determinants capable of predicting individual responsiveness to specific dietary components.

Among dietary bioactive compounds, polyphenols have attracted considerable interest because of their potential role in promoting human health. Polyphenols comprise a large and heterogeneous family of plant-derived compounds, including flavonoids, phenolic acids, stilbenes, lignans, and tannins. They are widely present in fruits, vegetables, legumes, whole grains, tea, coffee, cocoa, nuts, extra virgin olive oil, and red wine [3,4]. Mediterranean dietary patterns, characterized by a high intake of plant-based foods, extra virgin olive oil, fiber, and polyphenol-rich foods, have been shown to modulate gut microbiota composition and microbial metabolite production [5]. Epidemiological and intervention studies have associated polyphenol intake with beneficial effects on cardiometabolic health, endothelial function, glucose metabolism, oxidative balance, cognitive performance, and healthy aging [6,7,8]. Nevertheless, the magnitude and consistency of these effects differ markedly among individuals, suggesting that polyphenol intake alone is not sufficient to predict biological efficacy [9]. Importantly, these associations should be interpreted cautiously, as human intervention studies differ substantially in design, duration, dose, food matrix, background diet, study population, and outcome measures.

A key explanation for this variability lies in the limited bioavailability of many native polyphenols. Most dietary polyphenols are poorly absorbed in the small intestine because of their chemical complexity, glycosylation, polymerization, esterification, and interactions with the food matrix [3,10]. As a consequence, a substantial proportion of ingested polyphenols reaches the colon, where it becomes available for gut microbial metabolism. Through enzymatic reactions such as deglycosylation, dehydroxylation, demethylation, reduction, decarboxylation, and ring fission, intestinal microorganisms convert poorly absorbed polyphenols into a wide range of low-molecular-weight metabolites, including urolithins, enterolignans, phenyl-γ-valerolactones, phenolic acids, and equol [11,12,13,14].

The gut microbiota therefore acts as a metabolic interface that contributes to the biological fate of dietary polyphenols. Rather than being only a passive target of dietary modulation, the intestinal microbial ecosystem can transform complex dietary molecules into metabolites with distinct absorption profiles, tissue distribution, and biological activities [15,16]. In many cases, microbial-derived metabolites may be more relevant than parent compounds in mediating the physiological effects attributed to polyphenol-rich foods [11,17]. However, the relative contribution of parent compounds, microbial metabolites, and host phase-II conjugates remains incompletely defined and may differ across polyphenol classes, biological matrices, and clinical contexts. This concept has shifted the interpretation of polyphenol bioactivity from a compound-centered perspective toward a host–microbiota metabolic framework.

However, the microbial capacity to transform polyphenols is not uniformly distributed across individuals. Differences in gut microbiota composition, strain-level variation, microbial gene content, habitual diet, age, health status, medication exposure, and lifestyle factors may strongly influence the production of specific polyphenol-derived metabolites [9,18]. This has led to the concept of microbial metabotypes, defined as distinct metabolic phenotypes determined by the capacity of the gut microbiota to convert dietary substrates into specific microbial-derived metabolites [19]. Well-characterized examples include urolithin metabotypes derived from ellagitannin metabolism, equol producer and non-producer phenotypes following soy isoflavone intake, and interindividual differences in enterolignan production from dietary lignans [19,20,21,22]. Nevertheless, metabotype definitions are not equally standardized across polyphenol classes, and some proposed metabotypes currently represent emerging metabolic response patterns rather than validated clinical phenotypes.

The metabotype concept has important implications for precision nutrition. If the biological activity of polyphenols depends, at least in part, on microbial conversion, then individuals may respond differently to the same polyphenol-rich food or supplement according to their gut microbial metabolic profile. Identifying microbial metabotypes may therefore help improve responder stratification in nutritional studies and support the design of microbiome-informed dietary interventions [1,2,19]. However, metabotype-guided precision nutrition should not currently be interpreted as an established clinical tool. Its translation into routine dietary prescription is limited by heterogeneous dietary challenge protocols, non-standardized sampling time points and analytical cut-offs, incomplete evidence on metabotype stability, limited reproducibility across populations, and insufficient long-term validation in controlled human trials. These limitations are particularly relevant because metabolite production does not necessarily imply clinically meaningful benefit, and the relationship between metabotype status, systemic exposure, biological response, and health outcomes remains incompletely defined.

Recent reviews have provided broad overviews of polyphenol efficacy in relation to gut microbiota-mediated bioavailability, dose-dependent effects, gastrointestinal metabolism, health outcomes, and (poly)phenol-related gut metabotypes [23,24]. However, the specific role of microbial metabotypes as functional determinants of polyphenol bioactivity remains incompletely integrated into nutritional research and clinical translation. The novelty of the present review lies in integrating gut microbiota-mediated polyphenol biotransformation, metabotype classification, evidence hierarchy, methodological standardization, and translational barriers into a single critical framework. Rather than providing only a descriptive overview of polyphenol metabolism, this review distinguishes relatively well-characterized metabotype models, such as urolithin and equol production, from emerging or insufficiently standardized metabolic phenotypes, while evaluating their current readiness for precision nutrition applications. This distinction is important because classification systems differ in their biological readouts, analytical approaches, stability over time, and degree of human validation.

In addition, recent advances in metabolomics, metagenomics, metatranscriptomics, and computational modeling provide new opportunities to move metabotype research from descriptive classification toward functional prediction of host–microbiota interactions [25,26]. These approaches may help distinguish microbial metabolic potential from actual metabolite production and may improve the identification of metabolic responders and non-responders. However, their application requires harmonized analytical protocols, standardized reporting, external validation, and integration with clinically meaningful endpoints [27,28].

Therefore, this review aims to critically examine microbial metabotypes as functional determinants of polyphenol bioactivity, with particular emphasis on gut microbiota-mediated biotransformation, interindividual variability, evidence strength, methodological limitations, and translational potential. Particular attention is given to urolithin, equol, and enterolignan metabotypes, while also discussing emerging metabolic phenotypes, analytical challenges, multi-omics approaches, and the clinical gaps that currently limit the implementation of metabotype-guided precision nutrition.

## 2. Methods

This narrative review was developed through a structured targeted literature search focused on dietary polyphenols, gut microbiota-mediated biotransformation, microbial-derived metabolites, microbial metabotypes, and precision nutrition. Relevant articles published between 2004 and May 2026 were identified using PubMed/MEDLINE, Scopus, Web of Science, and Google Scholar, with the last search performed in May 2026. Search terms included combinations of “polyphenols”, “(poly)phenols”, “gut microbiota”, “microbial metabolism”, “polyphenol metabolites”, “microbial-derived metabolites”, “metabotypes”, “urolithins”, “equol”, “enterolignans”, “phenyl-γ-valerolactones”, “precision nutrition”, “personalized nutrition”, “metabolomics”, “metagenomics”, “metatranscriptomics”, “microbiome-guided nutrition”, and “predictive models”.

Peer-reviewed original articles, controlled human intervention studies, observational human studies, mechanistic animal studies, in vitro microbial metabolism studies, metabolomics studies, and recent reviews were considered. Articles were selected according to their relevance to at least one of the following themes: gut microbiota-mediated polyphenol biotransformation; production and quantification of microbial-derived metabolites; classification of microbial metabotypes; determinants of metabotype variability; and implications for precision nutrition. Priority was given to studies providing direct information on microbial metabolic phenotypes, validated metabolite measurements, controlled human intervention data, or mechanistic evidence explaining microbial biotransformation pathways.

Studies were excluded from the core discussion when they focused on polyphenols without considering microbial metabolism, did not report microbial-derived metabolites, did not address metabotype-based stratification or interindividual variability, or provided only general claims unrelated to gut microbiota-mediated polyphenol bioactivity. Human studies were prioritized when available, whereas animal, in vitro, and computational studies were included when they provided mechanistic insight into microbial biotransformation pathways, metabolite production, or biological activities of microbial-derived polyphenol metabolites. Additional references were identified from the bibliographies of relevant articles.

Evidence was interpreted according to study design and translational relevance. Controlled human intervention studies and studies using validated targeted metabolite measurements were considered the most directly relevant for human application. Observational studies were interpreted as supportive but not causal. Animal studies and in vitro experiments were used primarily to clarify mechanisms and were not considered sufficient to show clinical efficacy. Taxonomic associations between bacterial groups and metabolite production were distinguished from experimentally demonstrated microbial production or transformation.

Because this is a narrative review, no formal systematic review protocol, PRISMA-based study selection, risk-of-bias assessment, or meta-analysis was performed. This represents an inherent limitation, since narrative reviews may be affected by selection bias and do not provide the same reproducibility or formal grading of evidence as systematic reviews. Nevertheless, narrative reviews are useful for integrating heterogeneous evidence, developing conceptual frameworks, and identifying research gaps when a field includes mechanistic, clinical, analytical, and translational dimensions [29,30]. To improve transparency, the rationale for study selection, the hierarchy used to interpret evidence, and the limitations of the available data are explicitly described in this section and discussed throughout the manuscript.

### Evidence Appraisal and Interpretation Framework

The evidence supporting microbial metabotypes and their relevance to polyphenol bioactivity is heterogeneous and differs substantially across polyphenol classes, biological endpoints, and study designs [19,21,22,24,25]. Urolithin and equol metabotypes are supported by repeated human dietary challenge studies and validated metabolite measurements, although their direct predictive value for long-term clinical outcomes remains incompletely established [19,21,25]. Enterolignan-producing capacity is supported by human metabolite data and microbial consortium studies, but standardized cut-offs, dietary challenge protocols, and clinical outcome validation are less consistently available [22]. By contrast, metabolic phenotypes based on flavan-3-ol-derived phenyl-γ-valerolactones, anthocyanin-derived phenolic acids, or broad phenolic acid profiles remain emerging and should be interpreted as metabolic response patterns rather than clinically validated metabotypes [13,17,24].

Throughout this review, mechanistic plausibility is distinguished from clinical evidence. In vitro and animal studies are considered useful for pathway reconstruction, identification of microbial enzymes, and hypothesis generation, but they are not interpreted as proof of clinical benefit in humans [12,17,26]. Similarly, the presence or relative abundance of bacterial taxa associated with metabolite production is not considered sufficient evidence of causal metabolic function unless supported by isolation studies, enzymatic characterization, metagenomic functional data, or controlled dietary challenge with metabolite readouts [20,22,25,26]. This distinction is particularly important because microbial metabolism often depends on strain-level functionality, ecological context, and cooperative microbial networks rather than on the presence of a single bacterial genus or species [20,22,26].

For this reason, conclusions regarding metabotype-guided precision nutrition are presented cautiously. Metabolite production is interpreted as evidence of microbial metabolic capacity but not necessarily as evidence of clinically meaningful benefit [19,24]. A metabotype can therefore be considered more translationally mature when it fulfills several criteria: reproducible classification after a defined dietary challenge, validated analytical measurement of target metabolites, evidence of stability or clearly characterized variability over time, replication across populations, and association with physiologically or clinically meaningful outcomes in controlled human studies [24,25,27].

## 3. Gut Microbiota-Mediated Biotransformation of Dietary Polyphenols

### 3.1. From Dietary Intake to Colonic Availability

Dietary polyphenols are structurally heterogeneous compounds whose biological activity depends not only on their concentration in foods but also on their bioaccessibility, intestinal absorption, host metabolism, and microbial transformation. Although polyphenol-rich foods are widely consumed as part of healthy dietary patterns, many native polyphenols show limited absorption in the upper gastrointestinal tract because of their molecular size, glycosylation, polymerization, esterification, and interactions with the food matrix [1,3]. Consequently, a large proportion of ingested polyphenols reaches the colon, where it becomes available for microbial metabolism [11,17].

This colonic availability is particularly relevant for high-molecular-weight and complex polyphenols, including proanthocyanidins, ellagitannins, anthocyanins, flavan-3-ols, and lignans. These compounds are often poorly absorbed in their native form but can be transformed by the gut microbiota into smaller metabolites with different physicochemical properties and greater systemic availability [11,13,31]. As a result, the biological effects attributed to polyphenol-rich foods may depend more on microbial-derived metabolites than on the parent compounds originally ingested [9,11].

The gut microbiota therefore acts as a biochemical interface between dietary intake and host physiology. Through microbial biotransformation, poorly absorbed dietary polyphenols are converted into metabolites such as phenolic acids, phenyl-γ-valerolactones, urolithins, enterolignans, and equol, many of which can be absorbed, circulate systemically, and interact with host tissues [13,14,19,22]. This concept is central to the metabotype framework because the ability to generate these metabolites is not uniformly distributed across individuals.

### 3.2. Deglycosylation as a Gateway Reaction

Many dietary polyphenols occur naturally as glycosides, in which the aglycone is bound to sugar moieties such as glucose, rhamnose, galactose, arabinose, or rutinose. Glycosylation influences solubility, stability, intestinal transport, and susceptibility to enzymatic hydrolysis. Before many polyphenols can undergo further microbial degradation, the glycosidic bond must be cleaved to release the corresponding aglycone. This process, known as deglycosylation, represents one of the earliest and most important steps in polyphenol biotransformation [3,12].

Although some deglycosylation may occur in the small intestine through host enzymes, the gut microbiota provides a broader enzymatic repertoire. Several intestinal bacteria express β-glucosidases, α-rhamnosidases, β-galactosidases, and other carbohydrate-active enzymes able to hydrolyze glycosylated polyphenols [12,18,24]. Bacterial genera frequently associated with these activities include Bifidobacterium, Lactobacillus, Bacteroides, Enterococcus, Eubacterium, and members of the Clostridium clusters [12,18].

Deglycosylation is particularly relevant for flavonoids, including quercetin glycosides, flavanones, anthocyanins, and isoflavones. For example, soy isoflavone glycosides require hydrolysis before further microbial conversion of daidzein into metabolites such as dihydrodaidzein, *O*-desmethylangolensin, and equol [14,21]. Similarly, quercetin glycosides from onions, apples, and tea can be hydrolyzed into quercetin aglycone and subsequently degraded into smaller phenolic acids [12]. Thus, deglycosylation acts as a gateway reaction that determines whether specific dietary polyphenols become available for downstream microbial metabolism.

### 3.3. Ring Fission and Generation of Low-Molecular-Weight Metabolites

After deglycosylation, polyphenol aglycones can undergo further microbial reactions, including dehydroxylation, demethylation, reduction, decarboxylation, and aromatic ring fission. Ring fission is especially important because it converts complex phenolic structures into low-molecular-weight metabolites that can be more readily absorbed and distributed systemically [11,17,32].

Flavan-3-ols, such as catechin, epicatechin, and procyanidins from cocoa, tea, grapes, berries, and apples, are well-characterized examples of microbial ring fission. In the colon, these compounds are transformed into phenyl-γ-valerolactones and phenylvaleric acids, which are considered major microbial metabolites of flavan-3-ol intake [13]. These metabolites may undergo additional β-oxidation-like reactions, generating smaller phenolic acids such as hydroxyphenylpropionic, hydroxyphenylacetic, and hydroxybenzoic acids [13,17].

Anthocyanins, abundant in berries, red grapes, cherries, and other pigmented plant foods, are also extensively metabolized by intestinal microorganisms. Because of their limited stability and low absorption in native form, anthocyanins can be degraded into phenolic acids such as protocatechuic acid, vanillic acid, syringic acid, gallic acid, and other aromatic metabolites, depending on the structure of the parent anthocyanidin [33,34]. These microbial-derived compounds may contribute substantially to the systemic effects associated with anthocyanin-rich foods.

Ellagitannins and ellagic acid represent one of the clearest models of microbiota-dependent polyphenol metabolism. These compounds, found in pomegranate, walnuts, strawberries, raspberries, and other berries and nuts, are poorly absorbed in their native form. In the colon, selected gut bacteria convert ellagic acid into urolithins, a family of dibenzopyran-6-one derivatives with greater bioavailability than their precursors [19,20]. Importantly, urolithin production differs markedly among individuals, making this pathway one of the best-established examples of microbial metabotype variability [19].

### 3.4. Microbial Enzymatic Machinery

The conversion of dietary polyphenols into bioactive metabolites depends on a complex enzymatic machinery distributed across different members of the gut microbiota. The main enzymatic activities involved include glycosidases, esterases, tannases, reductases, demethylases, dehydroxylases, decarboxylases, and enzymes involved in aromatic ring cleavage [12,17,32].

Recent mechanistic reviews have clarified that gut bacterial polyphenol metabolism is driven by coordinated enzymatic reactions, including glycosidase-mediated deglycosylation, reductase-dependent C-ring modification, chalcone isomerase activity, phloretin hydrolase-mediated cleavage, hydroxycinnamate esterases, phenolic acid decarboxylases, and dehydroxylases involved in urolithin formation. These enzymatic activities explain how structurally diverse dietary polyphenols are converted into absorbable low-molecular-weight metabolites [32].

Glycosidases are essential for the initial hydrolysis of glycosylated flavonoids, isoflavones, and anthocyanins. Esterases and tannases participate in the degradation of hydrolysable tannins, releasing gallic acid and ellagic acid from gallotannins and ellagitannins. Reductases are central to isoflavone metabolism, particularly in the conversion of daidzein toward equol production. Demethylases and dehydroxylases contribute to the sequential transformation of lignans, flavonoids, and phenolic acids, whereas ring-cleavage enzymes allow for the degradation of complex aromatic structures into smaller metabolites [12,17,22,32].

Importantly, microbial function may be more informative than taxonomic composition alone. Individuals with similar microbial taxa may differ in their ability to produce specific polyphenol-derived metabolites if their microbial communities harbor different catabolic genes or enzymatic pathways [22,32]. This functional perspective is particularly relevant for precision nutrition because the prediction of polyphenol responsiveness may require the identification not only of bacterial taxa but also of microbial metabolic capacity.

### 3.5. Bacterial Taxa and Cooperative Metabolism

Although several bacterial taxa have been associated with polyphenol metabolism, many pathways require cooperative interactions among different microorganisms. Polyphenol biotransformation should therefore be interpreted as a network-based process rather than the activity of isolated bacterial species [22,32].

For ellagitannin metabolism, members of the family *Eggerthellaceae* are among the best-characterized urolithin-producing bacteria. Specific members of the gut microbiota have been implicated in sequential urolithin metabolism. While *Gordonibacter* species have been associated with the formation of urolithin intermediates, recent mechanistic evidence shows that diet-derived urolithin A can also be produced by a dehydroxylase encoded by human gut *Enterocloster* species [20]. Consistent with these mechanistic findings, a human walnut dietary intervention showed that walnut intake increased urinary urolithin metabolites and altered gut microbiota composition, with enrichment of Gordonibacter and other bacterial genera associated with urolithin A and isourolithin A production [33]. Additional *Eggerthellaceae* members, including *Ellagibacter* isourolithinifaciens, have also been isolated from human feces and associated with urolithin production, supporting the existence of specialized microbial functions involved in this pathway [34].

Isoflavone metabolism provides another clear example of taxon-dependent biotransformation. The conversion of daidzein into equol is restricted to a subset of individuals and depends on the presence of equol-producing bacteria. Reported equol-producing taxa include *Adlercreutzia equolifaciens*, *Slackia equolifaciens*, *Slackia isoflavoniconvertens*, *Asaccharobacter celatus*, and related members of the *Eggerthellaceae* family [14,21]. This restricted microbial capacity explains the distinction between equol producers and non-producers, one of the most widely recognized examples of microbial metabolic phenotyping.

Lignan metabolism also depends on cooperative bacterial activity. Plant lignans such as secoisolariciresinol, matairesinol, pinoresinol, and lariciresinol are converted into the mammalian enterolignans enterodiol and enterolactone through sequential microbial reactions. A multi-species bacterial consortium, including *Eggerthella* lenta and other gut bacteria, has been shown to participate in the cooperative bioactivation of plant lignans [22]. This illustrates how microbial cooperation can determine the generation of bioactive metabolites from polyphenol precursors.

For flavan-3-ols and procyanidins, the microbial networks involved are more complex and less completely defined. However, members of *Bacteroides*, *Eubacterium*, *Eggerthella*, *Flavonifractor*, *Lachnospiraceae*, and *Ruminococcaceae* have been associated with flavonoid degradation and the formation of phenyl-γ-valerolactones and related phenolic acids [12,13]. Similarly, anthocyanin metabolism appears to involve broad microbial consortia, including *Bifidobacterium*, *Lactobacillus*, *Bacteroides*, and *Enterococcus*, which contribute to deglycosylation and aromatic degradation [12,18,35].

**Figure 1:**
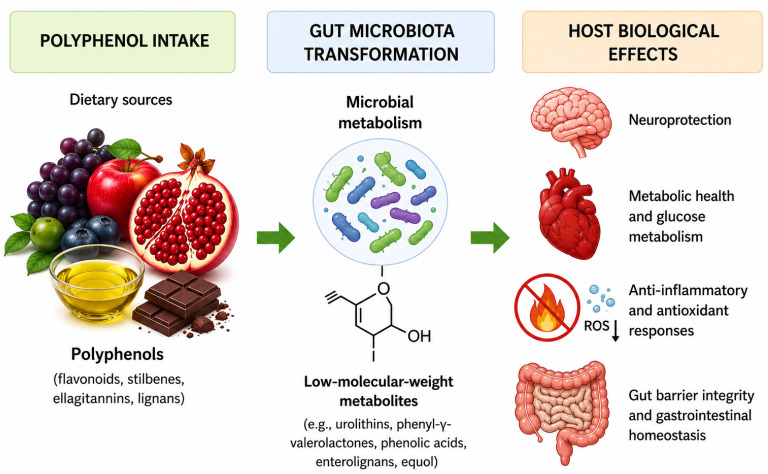
Gut microbiota-mediated transformation of dietary polyphenols and potential host biological effects. Dietary polyphenols from plant-based foods may reach the colon after limited upper gastrointestinal absorption and undergo gut microbiota-mediated metabolism. Microbial enzymatic activity can generate lower-molecular-weight metabolites, including urolithins, phenyl-γ-valerolactones, phenolic acids, enterolignans, and equol. These metabolites may influence several biological pathways, including metabolic regulation, inflammatory and oxidative stress responses, gut barrier function, endothelial homeostasis, and gut–brain communication. This figure is a simplified conceptual model created by the authors and does not imply linear causality or experimentally validated benefit in all contexts.

Overall, polyphenol biotransformation is a highly individualized microbial process. Differences in bacterial taxa, strain-level variation, enzymatic gene content, and cooperative metabolic networks determine whether an individual can efficiently generate specific bioactive metabolites. This functional heterogeneity provides the biological basis for microbial metabotypes and establishes a direct conceptual link between gut microbiota composition, polyphenol bioactivity, and precision nutrition. A schematic overview of the gut microbiota-mediated transformation of dietary polyphenols into bioactive metabolites and their downstream biological effects is shown in Figure 1.

Because the evidence linking specific bacterial taxa to polyphenol metabolite production differs substantially across pathways, Table 1 distinguishes experimentally implicated producers or transformers from associated taxa and inferred contributors. Evidence level and human validation status are also reported to avoid implying that all listed taxa have been causally demonstrated to produce the corresponding metabolites in humans.

## 4. Microbial Metabotypes

The concept of microbial metabotypes has emerged from the observation that individuals differ substantially in their ability to convert dietary polyphenols into specific bioactive metabolites. In this context, a metabotype can be defined as a functional metabolic phenotype determined by the enzymatic capacity of the gut microbiota to transform dietary substrates into characteristic microbial-derived metabolites. Unlike conventional taxonomic descriptions of the microbiota, microbial metabotypes focus on metabolic output, thereby providing a more direct link between diet, microbial function, metabolite bioavailability, and host responses [19,24].

This concept is particularly relevant for polyphenols because many of their health-related effects appear to depend on metabolites generated by the gut microbiota rather than on the parent compounds ingested with food [9,11,19]. Accordingly, (poly)phenol-related gut metabotypes have been proposed to classify individuals according to their capacity to generate specific microbial-derived metabolites, including urolithins, equol, lunularin, enterolignans, and phenyl-γ-valerolactones [24]. Thus, individuals consuming the same polyphenol-rich food may produce different metabolite profiles and, consequently, exhibit different biological responses. However, the strength of evidence supporting these metabotypes is not uniform: urolithin and equol metabotypes are relatively well characterized, whereas other proposed phenotypes remain less standardized and should be interpreted more cautiously.

Microbial metabotypes therefore offer a mechanistic framework to explain interindividual variability in polyphenol bioactivity, but their application to precision nutrition requires standardized classification protocols and clinical validation.

### 4.1. Urolithin Metabotypes

Urolithin metabotypes are among the best-characterized examples of microbiota-dependent polyphenol metabolism. Urolithins are produced from ellagitannins and ellagic acid, which are abundant in pomegranate, walnuts, strawberries, raspberries, and other berries and nuts. These parent compounds are poorly absorbed in the upper gastrointestinal tract and reach the colon, where specific gut bacteria convert them into urolithins through lactone-ring opening, decarboxylation, dehydroxylation, and related transformations [19,20,36].

Based on urinary and fecal urolithin profiles after ellagitannin or ellagic acid intake, individuals can be classified into three main urolithin metabotypes: urolithin metabotype A (UM-A), urolithin metabotype B (UM-B), and non-urolithin producer (UM-0). UM-A individuals mainly produce urolithin A; UM-B individuals produce urolithin A together with isourolithin A and/or urolithin B; whereas UM-0 individuals do not produce detectable urolithins [19,37]. This classification reflects differences in gut microbial metabolic capacity rather than differences in polyphenol intake alone. It should be acknowledged that urolithin metabotypes are operational classifications based on metabolite output after dietary challenge and may reflect dynamic functional states or production ratios rather than immutable host phenotypes.

The distribution of urolithin metabotypes appears to be associated with age, gut microbial composition, metabolic status, and possibly cardiometabolic risk. In this context, human evidence shows that gut microbial ecology associated with overweight and obesity can influence ellagic acid metabolism, supporting the view that host metabolic status and microbiota composition are important determinants of urolithin production and metabotype expression [38].

Some studies suggest that UM-A may be more often associated with healthier microbial and metabolic profiles, whereas UM-B has been linked to dysbiosis-prone signatures and less favorable cardiometabolic characteristics in some populations [19,37]. However, these associations are not yet definitive and should be interpreted cautiously, as urolithin production may be influenced by diet, health status, microbiota composition, and methodological differences in metabotype assessment.

Specific members of the gut microbiota, including *Eggerthellaceae*-related taxa, *Gordonibacter*, *Ellagibacter*, and *Enterocloster* species, have been implicated in urolithin production. Recent mechanistic evidence has identified an *Enterocloster*-encoded dehydroxylase involved in diet-derived urolithin A production, supporting a more detailed molecular basis for microbiota-dependent urolithin formation [20,34,36]. More recent evidence suggests that urolithin production may involve microbial consortia rather than single isolated species, supporting the view that metabotypes are functional ecosystem-level traits [39].

The urolithin model is particularly relevant for precision nutrition because it shows that the same dietary source of ellagitannins may lead to different circulating metabolites depending on the host microbiota. Therefore, assessing the urolithin metabotype may help identify individuals with different systemic exposure to urolithin-derived metabolites after ellagitannin intake. However, whether this classification consistently predicts clinically meaningful benefit remains to be established in long-term controlled human studies.

**Figure 2:**
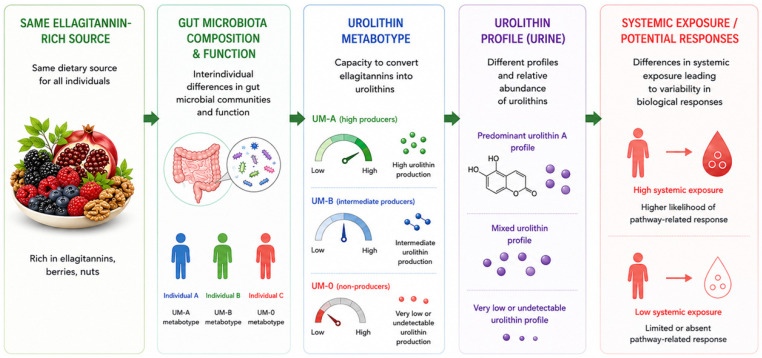
Interindividual variability in urolithin metabotype expression after ellagitannin intake. Following intake of ellagitannin- or ellagic acid-rich foods, individuals may differ in gut microbiota composition and functional capacity to convert these precursors into urolithins. These differences can define operational urolithin metabotype categories, including UM-A, UM-B, and UM-0. The figure illustrates possible differences in metabolite production and systemic exposure, but does not imply that urolithin metabotype status alone determines clinical outcome. This figure is a simplified conceptual model created by the authors.

Since urinary urolithin profiles reflect the individual capacity to generate and excrete microbial-derived metabolites, they may serve as practical readouts of systemic exposure to urolithins and may help explain differences in pathway-related biological responses after consumption of the same ellagitannin-rich foods. The interindividual variability in urolithin production and its relevance for metabotype classification are summarized in Figure 2.

### 4.2. Equol Producers and Non-Producers

Equol production is another paradigmatic example of microbial metabolic phenotyping. Equol is derived from daidzein, an isoflavone abundant in soy and soy-based foods. After ingestion, daidzein can be metabolized by intestinal bacteria through a series of reductive reactions leading to dihydrodaidzein, tetrahydrodaidzein, and finally equol [14,21]. However, only a subset of individuals possesses the microbial capacity to produce equol, leading to the classification of subjects as equol producers or non-producers.

Human intervention data in postmenopausal women further indicate that microbial metabolism of soy isoflavones may generate distinct metabolite patterns beyond the classical equol producer/non-producer classification, suggesting the existence of additional isoflavone-related metabotypes [45].

The prevalence of equol producers varies across populations and appears to be influenced by habitual diet, especially soy intake, as well as by gut microbiota composition. Equol-producing status is generally more common in populations with higher habitual soy consumption, although dietary intake alone does not fully explain this phenotype [14,21,40]. This indicates that both substrate availability and microbial enzymatic capacity are required for equol production.

Several bacterial taxa have been associated with equol formation, including *Adlercreutzia equolifaciens*, *Slackia equolifaciens*, *Slackia isoflavoniconvertens*, *Asaccharobacter* celatus, and related members of *Eggerthellaceae* [14,21]. A large Japanese population study reported that the prevalence and relative abundance of *Asaccharobacter celatus* and Slackia *isoflavoniconvertens* were higher in equol producers than in non-producers, suggesting that specific microbial signatures may contribute to this phenotype [40]. Nevertheless, the mere presence of equol-associated bacteria may not always guarantee equol production, indicating that strain-level functionality, microbial interactions, and ecological context are also important.

The equol producer phenotype has attracted considerable interest because equol displays higher estrogen receptor affinity and different biological properties compared with its precursor daidzein [14,21]. This has implications for the interpretation of soy isoflavone studies, in which inconsistent results may partly reflect differences in the proportion of equol producers among study populations. From a precision nutrition perspective, equol-producing status may help stratify individuals in soy isoflavone intervention studies; however, its predictive value for clinical outcomes depends on study population, endpoint, dose, background diet, and analytical definition of producer status [14,21,24].

### 4.3. Lignan Converters and Enterolignan Production

Plant lignans represent another class of polyphenols whose biological activity depends strongly on microbial metabolism. Lignans are found in flaxseed, sesame seeds, whole grains, legumes, fruits, and vegetables. After ingestion, plant lignans such as secoisolariciresinol diglucoside, matairesinol, pinoresinol, and lariciresinol are converted by the gut microbiota into the mammalian enterolignans enterodiol and enterolactone [22,43].

This conversion requires a sequence of microbial reactions, including deglycosylation, demethylation, dehydroxylation, and dehydrogenation. Importantly, no single bacterial species appears to perform the entire pathway independently. Instead, enterolignan production depends on cooperative metabolism involving different anaerobic bacteria, including *Eggerthella lenta*, *Blautia producta, Lactonifactor longoviformis*, and other gut taxa [22,43]. This makes lignan metabolism a clear example of microbial cooperation as a determinant of metabotype expression.

Interindividual variability in enterolignan production is substantial. Some individuals efficiently convert plant lignans into enterodiol and enterolactone, whereas others produce lower amounts despite similar dietary exposure. Recent microbiome-based studies have shown that enterolignan producers may have a distinct gut microbial composition compared with non-producers, with higher abundance of taxa belonging to *Ruminococcaceae* and *Lachnospiraceae* in some cohorts [44]. These findings support the possibility of showing microbial signatures predictive of lignan conversion capacity.

Enterolignan production is relevant because enterodiol and enterolactone have greater systemic availability than many plant lignan precursors and may contribute to the health effects associated with lignan-rich diets [22,43]. However, compared with urolithin and equol metabotypes, enterolignan-producing capacity is less consistently standardized in terms of dietary challenge, biological matrix, sampling time, and cut-off definition. Thus, lignan-converting capacity should currently be considered a promising but still incompletely standardized metabolic phenotype for future precision nutrition studies.

### 4.4. Metabotypes as Functional Biomarkers of Polyphenol Responsiveness

Taken together, urolithin metabotypes, equol-producing status, and lignan-converting capacity illustrate a common principle: the biological effects of polyphenols are partly determined by the metabolic competence of the gut microbiota. These metabotypes are not simply markers of microbial composition but functional readouts of microbial activity.

The postbiotic perspective further supports this concept, since microbial-derived polyphenol metabolites may represent key bioactive mediators linking gut microbiota-associated metabotypes with human health outcomes [46]. In this context, it has been proposed that the health effects of dietary polyphenols may be driven not only by the production of specific microbial metabolites but also by microbiota-associated metabotypes that reflect broader functional features of the intestinal ecosystem [47]. As such, metabotypes may provide more actionable information than taxonomic profiles alone when predicting individual responses to polyphenol-rich foods [19,24].

From a translational perspective, microbial metabotypes may serve as functional biomarkers to stratify individuals in nutritional intervention studies [19,24,46,47]. Their assessment could help distinguish metabolic responders from non-responders and explain heterogeneity in metabolite exposure and biomarker responses [19,24,25]. However, metabolic responder status should not be equated automatically with clinical benefit, and future studies should distinguish metabolite production from physiological or clinical responsiveness [19,24,47]. In the future, integrating metabotype classification with metagenomics, metabolomics, dietary assessment, and clinical phenotyping may support the development of microbiome-guided precision nutrition strategies, provided that these classifications are analytically standardized and clinically validated [25,26,27,28].

Recent evidence on metabotype clustering across resveratrol, daidzein, and ellagic acid metabolism further shows that combined metabotype profiles are associated with distinctive gut microbiome configurations and microbial interaction networks, supporting their potential value for functional stratification beyond single-compound metabotypes [48]. Nevertheless, combined metabotype clustering is still an emerging approach and requires replication across larger, independent, and geographically diverse cohorts before it can be used for clinical decision-making.

### 4.5. Standardization of Metabotype Assessment

A major limitation in the metabotype field is the lack of harmonized protocols for assigning individuals to specific microbial metabolic phenotypes [19,24,25]. Ideally, metabotype classification should be based on standardized dietary challenge tests using defined amounts of the relevant polyphenol precursor, controlled background diet, washout periods when appropriate, predefined biological matrices, validated analytical platforms, and explicit metabolite cut-off values [25,27,28]. Without such methodological standardization, metabotype categories may remain operational classifications that are difficult to compare across studies and populations [24,25,27,28].

For urolithin metabotypes, classification is commonly based on urinary or fecal urolithin profiles after consumption of ellagitannin- or ellagic acid-rich foods. For equol-producing status, urinary or plasma equol detection after soy isoflavone or daidzein exposure is frequently used. However, protocols differ substantially across studies with respect to dose, food matrix, intervention duration, washout period, timing of sample collection, analytical sensitivity, and criteria used to define producer and non-producer status. For equol-producing status, several operational definitions have been proposed, including urinary or serum equol concentrations and the urinary *S*-equol:daidzein ratio after soy challenge; the latter has been suggested to reduce variability related to isoflavone intake and pharmacokinetics [41,42]. Biological matrices also influence metabotype assignment. Urine is frequently used because it integrates metabolite production and excretion over time, whereas plasma or serum may better reflect systemic exposure at specific time points. Fecal samples can provide information on local intestinal metabolism and microbial metabolic activity, but they may not directly correspond to absorbed metabolite availability. Analytical approaches, including targeted liquid chromatography–tandem mass spectrometry, high-resolution metabolomics, and nuclear magnetic resonance spectroscopy, vary in sensitivity, metabolite coverage, and comparability across studies. Future metabotype studies should report dietary challenge dose, food matrix, background diet, washout period, sampling time points, biological matrix, analytical method, limit of detection, metabolite identification confidence, and classification criteria, in line with broader metabolomics reporting standards [27,49].

Stability over time is another important issue. Some metabotypes may remain relatively stable under habitual dietary and microbial conditions, whereas others may shift after dietary changes, antibiotic exposure, aging, disease, or microbiota-modulating interventions [19,24,37]. Therefore, repeated measurements may be needed to distinguish stable metabotype traits from transient metabolic states [24,25,37]. Future studies should also determine whether changes in metabotype status are accompanied by reproducible changes in systemic metabolite exposure and clinically meaningful outcomes [24,25,27,28]. Until such evidence is available, microbial metabotypes should be interpreted as functional biomarkers for research stratification rather than established diagnostic or clinical decision-making tools [19,24,25].

## 5. Determinants of Metabotype Variability

The determinants discussed below are supported by different levels of evidence. Diet, antibiotic exposure, and baseline gut microbiota composition have relatively strong biological plausibility and experimental support, whereas the specific contribution of host genetics and lifestyle factors to polyphenol-related metabotypes remains less directly established. Therefore, these determinants should be interpreted according to the strength and specificity of the available evidence. Microbial metabotypes are not fixed traits but dynamic phenotypes shaped by multiple host-related, environmental, dietary, and lifestyle factors. The capacity to convert polyphenols into specific bioactive metabolites depends on the presence of suitable microbial taxa, the availability of microbial enzymatic pathways, ecological interactions among gut bacteria, and repeated exposure to dietary substrates. Therefore, metabotype expression should be considered the result of a complex interaction between diet, gut microbial ecology, host biology, and environmental influences [9,19,24].

Understanding the determinants of metabotype variability is essential for translating the metabotype concept into precision nutrition. If individuals differ in their capacity to produce urolithins, equol, enterolignans, or other polyphenol-derived metabolites, then identifying the factors that modulate these capacities may help predict responders and non-responders to polyphenol-rich foods or supplements.

### 5.1. Diet

Diet is one of the strongest modulators of gut microbiota composition and function. Long-term dietary patterns influence microbial diversity, enterotype-like configurations, and the abundance of bacteria involved in carbohydrate, protein, lipid, and polyphenol metabolism [50,51]. Diets rich in plant-based foods, fiber, and polyphenols generally promote microbial communities with higher metabolic flexibility, whereas Western-style dietary patterns, typically high in saturated fats and refined carbohydrates, are associated with reduced microbial diversity and altered microbial functions [50,51,52].

For microbial metabotypes, diet acts through at least two mechanisms. First, it provides the polyphenol substrates required for metabolite production. For example, ellagitannin-rich foods such as pomegranate, walnuts, and berries are necessary precursors for urolithin production, while soy isoflavones are required for equol formation and lignan-rich foods such as flaxseed and whole grains support enterolignan production [19,21,22]. Second, habitual diet shapes the gut microbial ecosystem that determines whether these substrates can be converted into bioactive metabolites. Repeated exposure to specific polyphenol-rich foods may favor the expansion or functional activation of bacteria capable of metabolizing these compounds [9,24].

Short-term human intervention data also suggest that walnut consumption can modulate gut microbiota composition in a urolithin metabotype-dependent manner, supporting the role of both substrate exposure and baseline microbial metabolic capacity in shaping dietary responses [53].

However, substrate intake alone is not sufficient to guarantee metabolite production. Individuals may consume the same polyphenol source but produce different microbial metabolites depending on their microbial enzymatic capacity. This explains why dietary intervention studies often show heterogeneous responses, even when intake is controlled [24]. Therefore, diet should be interpreted both as a source of polyphenol precursors and as a long-term ecological pressure shaping metabotype expression.

### 5.2. Age

Age is another important determinant of gut microbiota structure and function. The gut microbiota changes substantially throughout life, from early colonization in infancy to progressive maturation during childhood and relative stabilization in adulthood, followed by further remodeling during aging [54,55]. These age-associated changes may influence microbial metabolic capacity, including the ability to transform dietary polyphenols into bioactive metabolites.

The relationship between age and polyphenol-related metabotypes has been particularly investigated in the context of urolithin production. Urolithin metabotype distribution appears to vary with age and physiological status, suggesting that the microbial functions required for ellagitannin metabolism may change across the lifespan [19,37]. In older adults, reduced microbial diversity, altered intestinal transit, medication use, dietary changes, and increased prevalence of chronic disease may all contribute to modified polyphenol metabolism [54,55].

Age-related changes in the gut microbiota may therefore affect both the quantity and type of microbial-derived polyphenol metabolites produced. This is relevant for precision nutrition because dietary recommendations based on polyphenol-rich foods may need to consider life stage, gut microbial maturity, and age-associated microbial functional decline.

### 5.3. Antibiotics and Medication Exposure

Antibiotics are among the most powerful disruptors of gut microbiota composition and function. Antibiotic exposure can reduce microbial diversity, eliminate specific taxa, alter microbial metabolic pathways, and produce long-lasting changes in community structure [56,57]. Because polyphenol metabotypes depend on specialized microbial functions, antibiotic-induced perturbations may impair the production of microbial-derived metabolites.

The impact of antibiotics is particularly relevant for metabotypes based on low-abundance or functionally specialized taxa, such as urolithin-producing or equol-producing bacteria. If these taxa are depleted or functionally suppressed, the individual’s ability to generate specific metabolites may be reduced or temporarily lost. Recovery after antibiotic exposure is highly individualized and may depend on baseline microbiota composition, diet, age, and repeated antibiotic use [56,57].

Other medications may also influence metabotype expression indirectly by changing gut microbiota composition, intestinal transit, bile acid metabolism, pH, or nutrient availability [56,57]. Although the effect of many non-antibiotic drugs on polyphenol metabotypes remains insufficiently characterized, medication exposure should be considered an important confounding factor in studies evaluating microbial metabolite production.

### 5.4. Geography and Ethnicity

Geographical location and ethnicity are strongly associated with gut microbiota composition, largely through differences in diet, lifestyle, sanitation, environment, cultural habits, and early-life exposures [52,58]. Population-level studies have shown that gut microbiome profiles differ across countries and regions, with diet and lifestyle often explaining a large proportion of the observed variation [52,58].

These geographical differences may influence microbial metabotype distribution. For example, equol producer prevalence differs markedly between populations and is generally higher in groups with habitual soy consumption, although microbial capacity remains essential for equol formation [14,21,40]. Similarly, differences in dietary exposure to ellagitannins or lignans, together with regional variation in gut microbiota composition, may influence urolithin and enterolignan production [19,22,24].

Geography should therefore not be interpreted only as a demographic variable but as a proxy for multiple ecological determinants of the gut microbiota. These include habitual diet, food availability, fermented food consumption, environmental microbial exposure, antibiotic use patterns, and lifestyle. For precision nutrition, this implies that metabotype-based recommendations may need to be validated in different populations rather than generalized from a single geographic cohort [52,58].

### 5.5. Host Genetics

Host genetics may contribute to gut microbiota variation by influencing immune function, mucosal glycosylation, intestinal barrier properties, bile acid metabolism, and nutrient handling. Population-based studies have shown that host-related factors may contribute to gut microbiota variation, although environmental factors often exert a stronger overall influence on microbiota composition than host genetics [59,60,61]. In the context of polyphenol metabotypes, host genetics may influence metabolite production indirectly by shaping the ecological environment in which specific microbial taxa persist. For instance, genetic variation affecting mucin composition, immune tolerance, or intestinal physiology may favor or limit colonization by bacteria involved in polyphenol biotransformation. However, current evidence suggests that metabotype expression is more directly determined by microbial functional capacity than by host genotype alone [19,24].

Therefore, host genetics should be considered one component of a broader host–microbiota–diet interaction. Future studies integrating genomics, metagenomics, and metabolomics may help clarify whether specific host genetic backgrounds predispose individuals to polyphenol-related metabotypes. However, direct evidence linking specific host genetic variants to defined polyphenol-related metabotypes remains limited. Most available data support an indirect role of host genetics through effects on gut microbial ecology rather than direct genetic determination of metabotype status [19,24,59,60,61].

### 5.6. Lifestyle

Lifestyle factors, including physical activity, sleep, stress, smoking, alcohol intake, and overall dietary behavior, can influence gut microbiota composition and function. Physical activity has been associated with increased microbial diversity and enrichment of taxa involved in short-chain fatty acid production and metabolic regulation, although these effects are often difficult to separate from dietary habits and body composition [62,63,64].

Exercise may indirectly influence polyphenol metabotypes by modifying microbial ecology, intestinal transit, systemic metabolism, and dietary choices [62,63,64]. Similarly, chronic stress and sleep disruption may alter gut microbial composition through neuroendocrine and autonomic pathways, potentially affecting microbial metabolic activity [50,52,58]. Smoking and excessive alcohol consumption can also reshape gut microbiota and intestinal physiology, although their specific impact on polyphenol metabotypes remains poorly defined [50,52,58].

Lifestyle should therefore be considered an important modulator of metabotype stability and responsiveness [19,24,37]. In precision nutrition, metabotype assessment may be most informative when combined with lifestyle profiling, including habitual diet, physical activity, medication history, and metabolic health status [1,2,24,50,62,63,64]. Although lifestyle factors are biologically plausible modulators of gut microbiota function, their specific effects on urolithin, equol, enterolignan, or other polyphenol-related metabotypes remain insufficiently characterized [19,24]. Many associations may be confounded by diet, medication use, adiposity, age, and health status [50,52,56,57,58].

### 5.7. Dynamic Nature of Microbial Metabotypes

Although metabotypes are often described as discrete categories, they should not be considered completely static. Some metabotypes may remain relatively stable over time, especially when supported by consistent dietary patterns and stable microbial communities. However, they may also be modified by dietary shifts, antibiotics, aging, disease, or lifestyle interventions [24,37].

This dynamic nature has important implications. First, metabotype classification should ideally be assessed under standardized dietary exposure to the relevant polyphenol precursor [24,25]. Second, repeated measurements may be necessary to distinguish stable metabotype traits from transient metabolic states [24,25,37]. Third, targeted dietary strategies, prebiotics, probiotics, or synbiotics could potentially shift microbial metabolism toward a more favorable metabolite-producing profile, although this remains an area requiring further investigation [24,50].

Overall, metabotype variability reflects the functional plasticity of the gut microbiome. This plasticity represents both a challenge and an opportunity for precision nutrition: while it complicates the prediction of individual responses, it also suggests that microbial metabolic capacity may be modifiable through targeted nutritional and lifestyle interventions [24,50,62,63,64].

## 6. Biological Activity of Microbial Polyphenol Metabolites

Microbial-derived polyphenol metabolites are increasingly recognized as potential mediators of the biological effects associated with polyphenol-rich foods. Compared with many parent polyphenols, these metabolites are generally smaller, more absorbable, and more likely to reach systemic circulation at physiologically relevant concentrations [11,13,17,24]. This is particularly important for compounds such as urolithins, phenyl-γ-valerolactones, phenolic acids, enterolignans, and equol, which are generated through gut microbial metabolism and may exert biological activities distinct from those of their dietary precursors [13,14,19,22].

However, the biological relevance of these metabolites should be interpreted according to the level of supporting evidence. For some metabolites, particularly urolithin A, human supplementation studies provide direct evidence for effects on biomarkers related to mitochondrial function and muscle physiology. By contrast, evidence for endothelial, neuroprotective, anti-inflammatory, gut barrier, and several metabolic effects often derives from in vitro experiments, animal models, short-term human studies, or associative metabolomics. Therefore, these mechanisms should be considered biologically plausible but not uniformly validated in humans.

The biological activity of microbial polyphenol metabolites should not be interpreted only through their antioxidant properties. Although redox modulation remains relevant, current evidence suggests that these compounds may act mainly as signaling molecules able to influence mitochondrial function, endothelial homeostasis, metabolic regulation, cellular stress responses, and neuroprotective pathways [6,13,65]. This signaling-oriented perspective is consistent with the metabotype concept because individuals with different microbial metabolic capacities may generate different circulating metabolites and may therefore show different physiological responses to the same polyphenol-rich food. Nevertheless, metabolite production should not be considered equivalent to clinical efficacy, and the relationship between microbial metabolite exposure and health outcomes requires validation in well-controlled human studies.

### 6.1. Mitochondrial Metabolism

Among microbial polyphenol metabolites, urolithin A is one of the best-characterized compounds in relation to mitochondrial function. Urolithin A is produced from ellagitannins and ellagic acid by specific gut microbial communities and has been identified as a natural inducer of mitophagy, the selective removal of damaged mitochondria [56]. In preclinical models, urolithin A promoted mitochondrial quality control, improved mitochondrial respiratory capacity, and enhanced muscle function during aging [56]. These preclinical findings provide mechanistic support for the mitochondrial effects of urolithin A, but they should not be interpreted as direct evidence of clinical efficacy.

Human studies support the translational relevance of this pathway, particularly for standardized urolithin A supplementation. In a first-in-human clinical study, urolithin A was reported to be safe and to induce molecular signatures related to mitochondrial and cellular health [66]. In older adults, urolithin A supplementation improved muscle endurance and modulated plasma biomarkers associated with mitochondrial health, including acylcarnitines and ceramides [67]. Similarly, a randomized trial in middle-aged adults showed improvements in muscle strength, exercise performance, and biomarkers of mitochondrial health after urolithin A supplementation [68]. However, these trials mainly evaluated direct urolithin A supplementation rather than endogenous urolithin production after habitual intake of ellagitannin-rich foods.

These findings suggest that microbial metabolites derived from polyphenols may influence host physiology by targeting mitochondrial quality control rather than simply acting as direct antioxidants. This is relevant for aging, metabolic dysfunction, and skeletal muscle decline, all of which are characterized by impaired mitochondrial efficiency and reduced cellular resilience. However, whether individuals capable of producing urolithin A after ellagitannin intake consistently experience comparable long-term mitochondrial or clinical benefits from dietary precursors alone remains uncertain. This distinction is important because endogenous metabolite production, supplement-derived exposure, and habitual food intake may differ substantially in dose, pharmacokinetics, and biological effects.

Other microbial-derived phenolic metabolites may also influence mitochondrial metabolism, although the evidence is less developed than for urolithin A. Phenolic acids and flavan-3-ol-derived valerolactones have been shown in experimental systems to modulate oxidative metabolism, cellular redox balance, and stress-response pathways [13,17,24]. Future studies are needed to clarify whether these effects occur at concentrations achievable through diet and whether specific microbial metabotypes predict mitochondrial outcomes in humans.

### 6.2. Endothelial Function

Endothelial function is another biological target potentially influenced by microbial polyphenol metabolites. The endothelium regulates vascular tone, platelet activity, leukocyte adhesion, and vascular homeostasis, largely through nitric oxide-dependent and nitric oxide-independent mechanisms. Polyphenol-rich foods have often been associated with improved vascular function, but the metabolites responsible for these effects are still being clarified [6,69]. Importantly, vascular responses to polyphenol-rich foods may reflect the combined influence of parent compounds, microbial metabolites, host phase-II conjugates, background diet, baseline cardiometabolic status, and medication use.

Urolithins have attracted attention as potential modulators of endothelial function. In vitro studies using human endothelial cells suggest that urolithin A, urolithin B, and conjugated urolithin metabolites may influence nitric oxide release and endothelial nitric oxide synthase activation [70]. These findings provide mechanistic plausibility, but they should be distinguished from direct clinical evidence. Moreover, a randomized clinical trial investigated urolithin A supplementation in individuals with relatively poor vascular endothelial function and reported that vascular responses were associated with gut microbiota features, supporting the concept that microbial metabolism and vascular physiology are interconnected [71]. However, this evidence remains limited and does not establish that urolithin metabotype status alone can predict vascular benefit after intake of ellagitannin-rich foods.

Flavan-3-ol-derived metabolites may also contribute to vascular effects. Phenyl-γ-valerolactones and phenylvaleric acids are major circulating microbial metabolites after flavan-3-ol intake and are considered relevant candidates for the vascular activity of cocoa, tea, apples, grapes, and berries [13]. Experimental studies indicate that colonic flavanol metabolites can stimulate nitric oxide production and protect endothelial cells under oxidative stress conditions [72]. These findings suggest that microbial conversion of flavan-3-ols may contribute to the vascular benefits associated with flavanol-rich foods. However, much of this evidence remains mechanistic or experimental, and the extent to which these metabolites mediate vascular effects at diet-achievable concentrations in humans remains uncertain.

Overall, the endothelial effects of microbial polyphenol metabolites remain promising but incompletely established [13,70,71,72]. Human data are available for selected interventions, but many mechanistic findings derive from in vitro endothelial models, sometimes using concentrations that may not fully reflect physiological exposure. Therefore, future human studies should integrate targeted metabolomics, standardized metabotype classification, vascular endpoints, and controlled dietary designs to determine whether specific microbial metabolite profiles causally contribute to endothelial benefits [13,70,71,72].

### 6.3. Metabolic Health

Microbial polyphenol metabolites may influence metabolic health through effects on glucose metabolism, lipid handling, energy homeostasis, mitochondrial function, and tissue-specific signaling pathways. In this context, polyphenols have been investigated for their potential role in obesity-related metabolic dysfunction, with evidence suggesting effects on adipogenesis, oxidative stress, lipid metabolism, cellular stress responses, and energy homeostasis [73]. This is particularly relevant for obesity, insulin resistance, metabolic syndrome, and age-related metabolic decline, where altered mitochondrial function and impaired metabolic flexibility are common features [1,6,65]. Polyphenol-rich foods have also been discussed as potential dietary tools for supporting glucose homeostasis and type 2 diabetes management, although interindividual variability in response remains a major limitation [74]. However, evidence linking specific microbial metabotypes to long-term metabolic outcomes remains heterogeneous and should be interpreted according to study design, duration, endpoint, and degree of metabolite validation.

A recent systematic review specifically examining (poly)phenol metabotypes and cardiometabolic health further supports the relevance of metabotype-based stratification for interpreting heterogeneous responses to polyphenol-rich foods and interventions [75]. Nevertheless, the current evidence base remains limited by variability in metabotype definitions, intervention protocols, biological matrices, and cardiometabolic endpoints.

Equol is a well-known example of a microbial-derived metabolite with potential metabolic relevance. Produced from daidzein by equol-producing bacteria, equol has biological properties distinct from those of its precursor, including higher affinity for estrogen receptors and endocrine-like effects [14,21]. These properties may partly explain why the metabolic and cardiometabolic effects of soy isoflavones vary between equol producers and non-producers [14]. Thus, equol-producing status may be an important determinant of individual response to soy-based dietary interventions. However, the clinical relevance of equol-producer status depends on the outcome considered and remains influenced by dose, background diet, population characteristics, and analytical definition of producer status.

Enterolignans, including enterodiol and enterolactone, are generated from plant lignans by cooperative gut microbial metabolism [22,43]. These metabolites may contribute to the biological effects associated with lignan-rich foods such as flaxseed, whole grains, legumes, fruits, and vegetables. Because enterolignan production varies substantially among individuals, lignan-converting capacity may represent another functional marker of metabolic responsiveness [22,44]. However, compared with urolithin and equol phenotypes, enterolignan-producing capacity is less consistently standardized and less directly validated as a predictor of cardiometabolic benefit.

Urolithin A may also contribute to metabolic health by improving mitochondrial quality control and cellular energy metabolism [65,66,67,68]. The relevance of this mechanism extends beyond muscle physiology, since mitochondrial dysfunction contributes to metabolic impairment in several tissues. However, whether endogenous urolithin-producing metabotypes display better long-term metabolic outcomes than non-producers remains insufficiently established and should be addressed in prospective human studies.

Overall, microbial polyphenol metabolites may act as metabolic modulators rather than classical nutrients. However, the strength of evidence differs substantially among metabolites and outcomes. Equol-producing status and urolithin production provide biologically plausible explanations for heterogeneous responses to soy isoflavones and ellagitannin-rich foods, but the extent to which these metabotypes predict long-term metabolic outcomes remains insufficiently established. Similarly, enterolignan-producing capacity may represent a functional marker of lignan metabolism, but standardized classification and clinical validation are still limited. Future studies should distinguish metabolic responsiveness, defined as production of target metabolites, from physiological responsiveness, defined as measurable improvement in glucose, lipid, inflammatory, vascular, or body-composition outcomes.

### 6.4. Neuroprotection

The gut–brain axis provides another relevant framework for understanding the biological activity of microbial polyphenol metabolites. Several microbial-derived phenolic compounds are small enough to cross biological barriers or influence peripheral pathways that indirectly affect brain function [13,17,76]. Potential mechanisms include modulation of mitochondrial function, neuronal stress responses, cerebrovascular function, synaptic plasticity, and gut–brain signaling. Previous evidence has also suggested that dietary polyphenols may support brain health through mechanisms involving redox balance, vascular function, neuronal resilience, and modulation of age-related neurobiological processes [77]. However, the contribution of microbial-derived metabolites to these effects remains difficult to isolate from the broader effects of dietary patterns, parent compounds, host conjugates, and vascular or metabolic improvements.

Urolithin A is again a particularly relevant candidate because of its ability to stimulate mitophagy and improve mitochondrial quality control [65]. Since mitochondrial dysfunction is involved in neurodegenerative processes, urolithin A has been investigated in experimental models of brain aging and Alzheimer’s disease. Preclinical studies suggest that urolithin A may reduce amyloid-related pathology, improve cognitive outcomes, and support neuronal homeostasis through mechanisms involving autophagy and mitochondrial function [78,79]. These findings provide mechanistic plausibility, but they remain predominantly preclinical and should not be interpreted as evidence that urolithin-producing metabotypes predict neuroprotective outcomes in humans.

Flavan-3-ol-derived phenyl-γ-valerolactones and phenylvaleric acids may also be relevant for brain health. These metabolites are increasingly considered important circulating biomarkers and potential bioactive mediators of flavan-3-ol intake [13]. Their possible contribution to cognitive function and healthy aging is an emerging area of investigation, particularly because parent flavan-3-ols are extensively metabolized before reaching systemic circulation [13,80]. However, direct human evidence linking these metabolite profiles to cognitive benefit remains limited, and standardized metabotype-based analyses are still lacking.

Phenolic acids derived from microbial metabolism of anthocyanins and other flavonoids may also participate in neuroprotective pathways. Compounds such as protocatechuic acid, vanillic acid, and related metabolites have been investigated for their ability to modulate oxidative stress, neuronal viability, and cellular stress responses in experimental systems [17,35]. Nevertheless, more human data are needed to determine whether specific microbial metabolite profiles are associated with measurable cognitive or neuroprotective outcomes.

From a precision nutrition perspective, neuroprotective responses to polyphenol-rich foods may depend not only on intake but also on the microbial capacity to generate metabolites capable of reaching or indirectly influencing the nervous system. However, this remains an emerging hypothesis rather than a clinically validated framework. Future studies should combine standardized cognitive endpoints, brain-relevant biomarkers, metabolomic profiling, microbial functional analysis, and metabotype classification before metabotype-based neuroprotective recommendations can be proposed.

### 6.5. Signaling Pathways and Systems-Level Effects

Microbial polyphenol metabolites may act through multiple signaling pathways rather than a single molecular target. This view is consistent with the concept that polyphenols may act as hormetic modulators, activating adaptive cellular responses rather than functioning only as direct antioxidants. Polyphenol-rich dietary patterns have also been discussed in the context of cellular aging, telomere dynamics, and stress-response pathways [8,81]. However, systems-level effects are difficult to attribute to individual microbial metabolites because dietary polyphenols generate complex mixtures of parent compounds, microbial metabolites, and host-derived conjugates that may act simultaneously.

Urolithin A influences pathways involved in mitophagy, mitochondrial biogenesis, cellular stress adaptation, and energy metabolism [65,66,67,68]. Equol can interact with estrogen receptor-mediated signaling, which may influence vascular, metabolic, and endocrine-related outcomes [14,21]. Enterolignans may also interact with hormone-related and metabolic pathways, although their effects depend on exposure levels and individual metabolic capacity [22,43]. These mechanisms are biologically plausible, but their clinical relevance varies according to metabolite, dose, biological matrix, tissue exposure, and population studied.

Phenyl-γ-valerolactones, phenylvaleric acids, and smaller phenolic acids may affect cellular signaling through modulation of redox-sensitive pathways, nitric oxide signaling, mitochondrial function, and transcriptional responses involved in metabolic homeostasis [13,17,24,72]. These pleiotropic effects suggest that microbial metabolites can influence host physiology at several biological levels simultaneously. Nevertheless, much of the available evidence remains mechanistic, and future studies should determine whether these pathways are activated at physiologically achievable concentrations in humans.

This systems-level activity is central to the concept of microbial metabotypes. The biological response to a polyphenol-rich food is not determined only by the chemical composition of the food but also by the microbial capacity to generate a specific metabolite profile, host absorption and conjugation, tissue exposure, and baseline physiological status. Therefore, two individuals consuming the same food may generate different metabolite profiles and potentially activate different signaling pathways depending on their metabotype. However, this should be interpreted as a mechanistic framework rather than proof that metabotype classification alone can predict clinical benefit. The translational value of this framework will depend on future studies demonstrating that specific microbial metabolite profiles reproducibly predict clinically meaningful outcomes beyond general dietary intake and microbiome composition.

To summarize the heterogeneous evidence supporting the biological activity of microbial-derived polyphenol metabolites, Table 2 reports the main metabolite classes, proposed biological targets, level of supporting evidence, and current translational limitations. This distinction is important because several mechanisms are biologically plausible but not yet uniformly validated in controlled human studies.

## 7. Precision Nutrition

Precision nutrition aims to move beyond generalized dietary recommendations by accounting for the biological, metabolic, microbial, genetic, behavioral, and environmental characteristics that determine individual responses to foods and bioactive compounds. In the context of polyphenols, this approach is particularly relevant because their biological activity depends not only on dietary intake but also on gut microbial biotransformation, metabolite bioavailability, host conjugation, and metabotype-specific metabolic capacity [1,2,19,24]. Therefore, microbial metabotypes may represent functional biomarkers for stratifying individuals according to their ability to generate microbial-derived metabolites from polyphenol-rich foods. However, their use in precision nutrition remains at an early stage and should currently be considered a research and trial-stratification framework rather than a routine clinical tool.

The integration of metabotype assessment into precision nutrition could help explain why some individuals show clear physiological benefits after polyphenol intake, whereas others show limited or no response. This is especially relevant for ellagitannin-rich foods, soy isoflavones, lignan-rich foods, and flavan-3-ol-containing products, where microbial metabolism strongly influences systemic metabolite exposure [13,14,19,22]. Nevertheless, the prediction of responders and non-responders cannot rely on metabolite production alone. Clinical translation requires standardized dietary challenge protocols, validated metabolite cut-offs, reproducible classification across populations, and evidence that metabotype status improves prediction of meaningful outcomes beyond conventional dietary and clinical variables [24,25,27,28,49,75,86].

### 7.1. Responder and Non-Responder Phenotypes

A major challenge in polyphenol research is the heterogeneity of human responses to dietary interventions. This issue is particularly relevant for cardiometabolic outcomes, where systematic evidence indicates that (poly)phenol metabotypes may contribute to variability in metabolic and vascular responses [75]. Even when polyphenol intake is standardized, individuals may differ markedly in circulating metabolite concentrations, urinary excretion profiles, and physiological outcomes [19,24,87]. This variability has led to the classification of individuals as responders or non-responders, although these categories may vary depending on the metabolite measured, the biological endpoint considered, the analytical matrix, the duration of the intervention, and the criteria used to define response.

Microbial metabotypes provide a mechanistic explanation for at least part of this heterogeneity. Responder phenotypes may depend on both metabolite exposure and the underlying gut microbial ecology associated with specific metabotypes [47,48]. For example, individuals classified as UM-A, UM-B, or UM-0 after ellagitannin intake generate different urolithin profiles and may therefore differ in their systemic exposure to urolithin A, isourolithin A, or urolithin B [19,31]. Similarly, equol producers may respond differently to soy isoflavones than non-producers because equol has distinct biological properties compared with daidzein [14,21]. Lignan converters may also differ in their ability to generate enterodiol and enterolactone from plant lignans, potentially influencing the physiological effects of lignan-rich foods [22,44]. However, these examples mainly support differences in metabolic exposure; whether they consistently predict clinical benefit remains dependent on endpoint, dose, population, and study design.

A recent randomized crossover trial in postmenopausal women supports this responder/non-responder framework. After the intake of a polyphenol-rich mixture containing resveratrol, pomegranate ellagitannins, ellagic acid, and red clover isoflavones, clinically meaningful improvements in quality-of-life domains were mainly observed in equol producers and in specific metabotype clusters, suggesting that microbial metabolic capacity can influence the efficacy of polyphenol-based interventions [82]. Metabotype clustering across different polyphenol classes may better reflect real-life dietary exposure, where multiple polyphenols are consumed simultaneously, and may provide a more informative basis for microbiome-guided precision nutrition [24]. However, such findings require replication in larger, independent, and more diverse cohorts before they can support generalizable dietary recommendations.

Responder status should not be considered a simple binary trait. An individual may be a responder for one polyphenol class and a non-responder for another, depending on the microbial pathways involved. Moreover, response may be defined at different levels, including metabolite production, biomarker change, clinical outcome, or subjective health improvement. Therefore, future studies should distinguish between metabolic responders, who produce target microbial metabolites, and physiological responders, who show measurable improvements in health-related endpoints. This distinction is essential because metabolite production is necessary for exposure to some microbial-derived compounds but is not sufficient to demonstrate health benefit [19,24,75,82].

### 7.2. Microbiome-Guided Interventions

Microbiome-guided interventions represent a promising but still experimental strategy to optimize the effects of polyphenol-rich foods. In this framework, gut microbiota composition and function are assessed before intervention to identify individuals with the microbial capacity to metabolize specific polyphenol substrates. This could allow dietary recommendations to be tailored according to microbial metabolic potential rather than based only on food composition or population-level evidence [1,2,83]. However, current evidence is not yet sufficient to support routine microbiome-guided prescription of polyphenol-rich foods in clinical practice.

One possible approach is to use metabotype screening before recommending specific polyphenol-rich foods. For example, individuals able to produce urolithin A may be suitable candidates for interventions based on pomegranate, walnuts, or berries, whereas UM-0 individuals may require alternative strategies to enhance urolithin production or may benefit from direct supplementation with postbiotic metabolites. Similarly, equol-producing status may help identify individuals who may respond differently to soy isoflavone interventions [14,21]. Nevertheless, these strategies remain hypothetical until controlled trials demonstrate that metabotype-guided allocation improves clinical or physiological outcomes compared with non-stratified dietary advice [24,75,86].

Microbiome-guided interventions may also aim to modify microbial metabolism itself. Dietary strategies that increase microbial diversity, provide fermentable substrates, or repeatedly expose the gut ecosystem to selected polyphenols may support the growth or activity of metabolite-producing bacteria [50,51]. In the future, combining polyphenols with prebiotics, probiotics, or synbiotics could be used to enhance the production of specific metabolites, although the evidence for stable and predictable metabotype conversion remains limited [22,24,88,89]. Moreover, inducing metabolite production does not necessarily ensure sustained systemic exposure or clinical benefit, particularly if the ecological changes are transient or context-dependent.

An important methodological issue is that taxonomic profiling alone may be insufficient to guide interventions. Because metabolite production depends on microbial genes, enzymes, strain-level functionality, and ecological cooperation, functional approaches such as metagenomics, metatranscriptomics, and targeted metabolomics may provide more actionable information than bacterial abundance alone [22,26,83]. Therefore, microbiome-guided polyphenol interventions should ideally integrate microbial composition, microbial function, metabolite readouts, dietary assessment, and clinically meaningful endpoints.

### 7.3. Personalized Supplementation

Personalized supplementation represents a potential extension of metabotype-guided precision nutrition, but it remains an emerging strategy rather than an established clinical practice. Conventional supplementation strategies generally provide the same polyphenol source or dose to all individuals, despite substantial differences in intestinal absorption, microbial conversion, metabolite production, and physiological responsiveness. For polyphenols, this approach may be inadequate because the biological activity of many compounds depends on whether the gut microbiota can transform dietary precursors into bioactive microbial-derived metabolites [19,24].

A metabotype-informed strategy could, in principle, help select the most appropriate formulation for each individual. Subjects with efficient microbial conversion capacity may benefit from precursor-rich extracts, such as ellagitannin-containing products in urolithin producers or soy isoflavone preparations in equol producers [14,19,21,24]. Conversely, individuals with low or absent metabolite production may require alternative approaches, including direct supplementation with microbial-derived metabolites, longer dietary adaptation periods, or co-administration with microbiota-modulating compounds [19,24,66,67,68,90]. However, these strategies should be regarded as hypotheses requiring direct comparison in controlled trials rather than as validated supplementation algorithms.

Urolithin A provides the clearest example of this concept. Because not all individuals produce urolithin A after intake of ellagitannin-rich foods, direct supplementation with urolithin A has been investigated to bypass microbial conversion and provide standardized systemic exposure to the active metabolite [66,67,68]. Similar biomarker-guided approaches may be developed for other polyphenol-derived metabolites, but clinical evidence remains limited [90]. Future trials should determine whether metabotype-guided supplementation improves biological or clinical outcomes compared with conventional, non-stratified supplementation [24,75,91]. These trials should also evaluate dose–response relationships, long-term safety, population specificity, cost-effectiveness, and whether direct metabolite supplementation reproduces or differs from the biological effects of precursor-rich foods.

### 7.4. Artificial Intelligence and Predictive Models

Artificial intelligence (AI) and machine learning are increasingly used in precision nutrition to integrate high-dimensional data and predict individual dietary responses. These models can combine dietary intake, microbiome composition, metabolomics, clinical biomarkers, anthropometric data, physical activity, and lifestyle information to generate personalized recommendations [1,2,92]. A landmark study demonstrated that machine-learning models incorporating gut microbiota and host parameters could predict individualized postprandial glycemic responses to meals, providing proof of concept for data-driven personalized nutrition [2]. However, prediction of glycemic responses cannot be directly extrapolated to polyphenol metabotypes without dedicated validation studies.

In polyphenol research, AI-based models could be used to predict metabotype status, metabolite production, and physiological response to specific polyphenol-rich foods. For example, models could integrate baseline microbiome features, microbial gene content, habitual diet, age, medication exposure, and metabolomic signatures to estimate the probability of producing urolithins, equol, enterolignans, or flavan-3-ol-derived valerolactones. Such approaches could help identify potential responders before intervention and reduce heterogeneity in clinical trials. Nevertheless, these applications remain largely prospective, and their accuracy depends on the availability of standardized challenge tests, harmonized metabolomics, and well-characterized clinical endpoints.

AI may also support the design of personalized polyphenol interventions by identifying optimal food combinations, supplementation strategies, and microbial targets. For example, machine-learning algorithms could be trained to predict which dietary patterns favor metabolite-producing microbial networks or which individuals may require direct metabolite supplementation rather than precursor intake. Deep learning approaches have been proposed as particularly useful for microbiome-informed precision nutrition because they can capture complex nonlinear relationships between microbial communities, metabolites, and host responses [91]. However, interpretability remains a major challenge, especially when models identify statistical predictors without clarifying causal mechanisms.

Despite this potential, several challenges remain: Predictive models require large, well-characterized datasets with standardized dietary assessment, microbiome profiling, metabolomics, and clinical outcomes. In addition, models must be externally validated across populations, ethnic groups, age ranges, and dietary contexts. Issues related to data privacy, algorithmic bias, interpretability, regulatory oversight, and clinical implementation must also be addressed before AI-guided polyphenol recommendations can be translated into routine practice [86,92]. At present, AI-based prediction should be considered a tool for hypothesis generation, trial stratification, and model development rather than a validated basis for individualized polyphenol prescription.

### 7.5. Toward Metabotype-Guided Precision Nutrition

The future application of microbial metabotypes in precision nutrition will require a shift from food-based recommendations alone toward function-based dietary strategies. Rather than asking only whether a food contains polyphenols, precision nutrition should also ask whether an individual can transform those polyphenols into microbial-derived metabolites, whether these metabolites reach systemic circulation, and whether they are linked to meaningful physiological or clinical outcomes [1,2,19,24,75,84].

A practical metabotype-guided framework could include four steps: first, identification of the relevant polyphenol precursor and expected microbial metabolites; second, assessment of baseline microbial and metabolomic profiles; third, classification of individuals according to metabotype or metabolite-producing capacity; and fourth, selection of personalized interventions, including dietary sources, precursor supplements, postbiotic metabolites, or microbiota-modulating strategies [19,24,25,83,84]. However, each of these steps requires methodological standardization before clinical implementation. In particular, future studies should harmonize dietary challenge protocols, biological matrices, sampling time points, analytical platforms, metabolite cut-offs, and criteria for defining response [25,27,28,49].

This approach may improve the design of future clinical trials by reducing biological heterogeneity and enabling stratified analyses. It may also support more precise dietary recommendations for consumers, patients, and specific population groups [24,25,75,82,86]. However, metabotype-guided nutrition should be developed cautiously, with strong evidence from controlled human studies and validated biomarkers [75,84,86,90]. At present, the metabotype concept is promising but still requires standardization, replication, and clinical outcome validation before broad application. Therefore, metabotype-guided recommendations should currently be considered a research-informed framework rather than a routine clinical tool [24,75,84,86].

**Figure 3:**
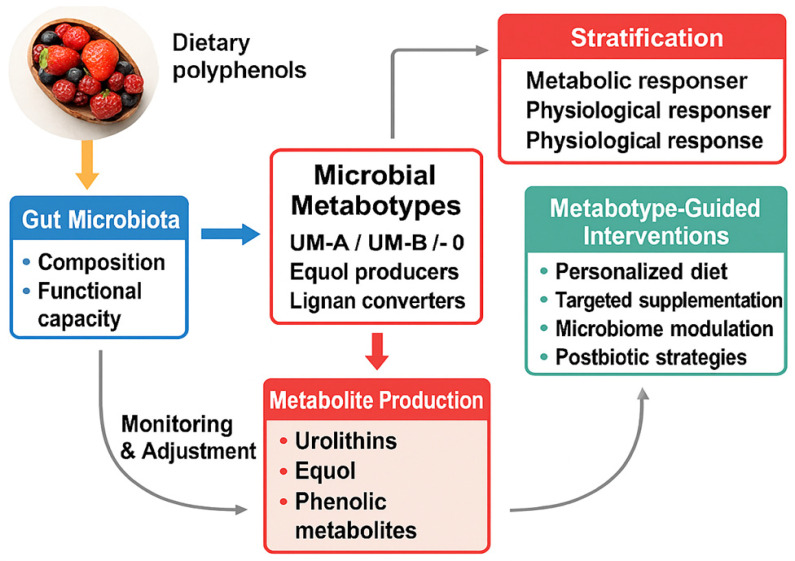
Proposed workflow for integrating microbial metabotype assessment into precision nutrition strategies. A metabotype-guided precision nutrition workflow may include identification of the relevant polyphenol precursor, controlled dietary challenge, assessment of gut microbiota composition and function, metabolomic measurement of microbial-derived metabolites, metabotype classification, and evaluation of physiological or clinical responses. These data may support responder stratification and the design of personalized dietary or supplementation strategies. This workflow is a proposed research framework and should not be interpreted as a validated clinical algorithm for routine dietary prescription.

Overall, microbial metabotypes provide a mechanistic bridge between polyphenol intake and individualized biological response. Their integration with metabolomics, microbiome science, and AI-based predictive modeling may represent a useful step toward stratified nutritional strategies, provided that these approaches are externally validated, clinically interpretable, and shown to improve outcomes beyond standard dietary assessment [1,2,26,83,84,85,86,91,92]. A proposed workflow for integrating microbial metabotype assessment into precision nutrition strategies is shown in Figure 3.

## 8. Future Perspectives

The metabotype concept provides a promising framework for understanding interindividual variability in polyphenol bioactivity, but its translation into clinical and nutritional practice remains at an early stage. Future research should move beyond descriptive associations between polyphenol intake, gut microbiota composition, and health outcomes toward function-based strategies capable of identifying, predicting, and potentially modulating microbial metabolic capacity. In this context, microbiome engineering, next-generation probiotics, metabolomics, and personalized nutraceuticals represent key future directions. However, these approaches should be considered research priorities rather than clinically established interventions, because their feasibility, safety, reproducibility, regulatory classification, and long-term efficacy remain incompletely defined.

### 8.1. Microbiome Engineering

Microbiome engineering refers to strategies designed to modify gut microbial composition or function in a targeted manner. In the context of polyphenol metabolism, the goal would not simply be to increase microbial diversity, but to enhance specific microbial functions required for the production of bioactive metabolites, such as urolithins, equol, enterolignans, and flavan-3-ol-derived valerolactones [19,22,26]. However, the ability to deliberately and reproducibly modify polyphenol-related metabotypes in humans remains largely unproven.

Potential approaches include dietary modulation, targeted prebiotics, synbiotics, defined microbial consortia, and engineered live biotherapeutics. For example, individuals classified as UM-0 after ellagitannin intake may theoretically benefit from strategies aimed at promoting urolithin-producing microbial networks. In vitro confirmatory approaches have shown that selected bacterial strains, such as Streptococcus thermophilus, may enhance urolithin A yield when combined with human gut microbiota from different urolithin metabotypes, supporting the feasibility of microbiota-targeted strategies to improve urolithin production [88]. Similarly, non-equol producers may require interventions designed to support the colonization or functional activation of equol-producing bacteria. However, such approaches remain largely experimental and require rigorous validation in controlled human studies before they can be considered practical nutritional strategies.

A major challenge is that polyphenol metabolism often depends on microbial cooperation rather than on a single bacterial strain. Therefore, successful microbiome engineering may require the design of microbial consortia with complementary enzymatic functions, rather than supplementation with isolated microorganisms [22,26]. This is especially relevant for lignan conversion, where enterolignan production depends on sequential reactions performed by different bacterial taxa [22]. In addition, engraftment, ecological stability, substrate availability, interindividual microbiome resistance, and diet–microbe interactions may all influence whether engineered or supplemented microbial functions persist in vivo.

Synthetic biology may further expand this field by enabling the development of engineered microorganisms capable of sensing gut environmental signals, producing specific metabolites, or enhancing substrate conversion [89,93,94]. However, microbiome engineering for nutritional purposes must address several unresolved issues, including safety, ecological stability, colonization resistance, off-target metabolic effects, horizontal gene transfer, regulatory classification, and long-term persistence in the gut ecosystem. For this reason, microbiome engineering should currently be viewed as an experimental research direction rather than an immediately translatable approach for metabotype-guided precision nutrition.

### 8.2. Next-Generation Probiotics

Next-generation probiotics differ from traditional probiotic strains because they are often selected on the basis of specific functional properties, mechanistic evidence, and disease- or metabolism-related targets. In the context of polyphenol metabotypes, next-generation probiotics could be selected for their ability to perform defined biotransformation reactions or to support microbial networks involved in polyphenol metabolism [89]. However, the development of next-generation probiotics for metabotype modulation remains at an early stage and requires validation of efficacy, safety, colonization capacity, and long-term functional persistence.

Candidate organisms may include taxa involved in urolithin, equol, or enterolignan production, such as members of *Eggerthellaceae*, *Gordonibacter*, *Ellagibacter*, *Adlercreutzia*, *Slackia*, *Asaccharobacter*, *Eggerthella*, and *Lactonifactor* [20,21,22,34]. However, the development of such probiotics is complex. Some of these taxa are strict anaerobes, may be difficult to culture, may require specific ecological partners, and require careful safety evaluation before human use. Moreover, the presence of a candidate metabolite-producing organism does not necessarily guarantee metabolite production in vivo, because expression of the relevant pathways may depend on strain-level functionality, substrate availability, gut ecological context, and host factors.

Another important issue is whether administration of a single metabolite-producing strain is sufficient to change an individual’s metabotype. Since polyphenol metabolism often depends on ecological cooperation, future interventions may need to use defined consortia rather than single strains [22,32,39]. These consortia should be designed to reproduce complete metabolic pathways, from precursor degradation to final bioactive metabolite production [22,32]. Even in this case, durable metabotype conversion would need to be demonstrated through repeated dietary challenge tests, metabolite measurements, and follow-up after supplementation withdrawal [25,27,89].

Next-generation probiotics could also be combined with specific polyphenol substrates to create targeted synbiotic formulations. For example, a urolithin-oriented synbiotic could theoretically combine ellagitannin-rich extracts with bacteria capable of supporting urolithin production [19,20,36,39,88]. Similarly, soy isoflavones could be combined with equol-producing strains or microbial consortia [14,21,40,41,42]. Nevertheless, clinical trials are needed to determine whether such approaches can induce stable metabotype conversion, increase systemic metabolite exposure, and translate into measurable health benefits [25,75,86,89]. Until such evidence is available, next-generation probiotics for polyphenol metabotype modulation should be regarded as experimental tools rather than validated precision nutrition interventions [86,89].

### 8.3. Metabolomics

Metabolomics will be essential for advancing the metabotype concept from theoretical classification to practical application. While microbiome sequencing provides information on microbial composition and potential function, metabolomics directly measures the biochemical output of host–microbe interactions. This is particularly important for polyphenols because the biological relevance of microbial metabolism depends on the actual production, absorption, circulation, conjugation, and excretion of metabolites [13,19,32].

Targeted metabolomics can quantify specific metabolites, such as urolithin A, urolithin B, equol, enterolactone, enterodiol, phenyl-γ-valerolactones, and phenolic acids. Systematic evidence indicates that profiling endogenous and gut microbial metabolites can help identify metabotype-specific dietary responses, supporting the use of metabolomics as a functional tool for stratifying individuals in precision nutrition studies [84]. This approach is useful for classifying individuals into metabotypes and assessing whether a dietary intervention successfully increases metabolite production. Untargeted metabolomics, on the other hand, may identify novel microbial-derived metabolites and unexpected metabolic signatures associated with polyphenol intake [83,85]. However, untargeted approaches require careful metabolite annotation, quality control, and validation, because unidentified or putatively annotated features may be difficult to translate into actionable metabotype classifications.

Beyond polyphenol research, fecal metabolic phenotyping based on ^1^H NMR spectroscopy has been used to define disease-associated metabotypes reflecting perturbations in gut microbial–host co-metabolism, supporting the broader utility of metabolomics for functional stratification of individuals [95]. Nevertheless, fecal metabolomics, urinary metabolomics, and plasma or serum metabolomics provide complementary but non-equivalent information: fecal profiles reflect luminal metabolism, urine integrates metabolite production and excretion over time, and plasma or serum better captures systemic exposure at specific sampling points. Therefore, metabotype classification may vary depending on the biological matrix used.

Future studies should integrate dietary assessment, gut microbiome profiling, and metabolomics in the same individuals [26,83,84,85]. This multi-omics approach may help distinguish between individuals who consume polyphenol-rich foods but fail to produce target microbial metabolites and those who generate high systemic metabolite exposure. Such a distinction is crucial for identifying true metabolic responders. However, metabolic responder status should be linked to biological or clinical endpoints before being used to guide personalized nutrition.

Standardization will also be important. Different studies currently use different biological matrices, including urine, plasma, serum, feces, and sometimes tissue samples. Timing of sample collection, analytical platforms, metabolite annotation, internal standards, data normalization, and dietary standardization can strongly influence results. Therefore, future metabotype studies should adopt harmonized protocols to improve reproducibility and comparability across populations [27,28]. At minimum, studies should report dietary challenge composition, dose, washout conditions, sampling time points, analytical method, limits of detection and quantification, metabolite identification confidence, and criteria used for metabotype assignments.

### 8.4. Personalized Nutraceuticals

Personalized nutraceuticals represent a potential future translational direction for metabotype-based nutrition, but they remain an emerging research and development area rather than an established clinical practice. Rather than simply adapting supplement choice to current metabotype status, the next generation of nutraceuticals may integrate polyphenol precursors, microbial-derived metabolites, microbiota-modulating components, and biomarker-based monitoring into targeted formulations [19,24,76,79].

In this framework, nutraceutical products could be developed according to defined metabolic objectives. For example, one formulation could aim to increase exposure to a specific microbial-derived metabolite, while another could be designed to support the microbial pathways required for its endogenous production. This distinction is important because some individuals may benefit from precursor-based approaches, while others may require direct metabolite delivery or synbiotic combinations intended to enhance microbial conversion [19,24,76]. However, such strategies require direct testing against conventional non-stratified supplementation approaches before they can be considered clinically useful.

The urolithin A model illustrates the feasibility of metabolite-based nutraceutical development, but broader application to other microbial polyphenol metabolites will require careful evaluation of safety, bioavailability, dose–response relationships, pharmacokinetics, long-term efficacy, and interindividual variability [57,58,59,76,79]. Future personalized nutraceuticals should also consider age, sex, habitual diet, medication use, metabolic health, and lifestyle, since these factors influence both gut microbial function and host metabolism [40,55]. Importantly, direct supplementation with a microbial-derived metabolite may not reproduce the full biological effects of the corresponding polyphenol-rich food, which contains multiple bioactive compounds and may influence the microbiota through additional substrates.

A key priority will be to move from generic plant-extract formulations toward evidence-based products supported by validated biomarkers. Ideally, personalized nutraceutical recommendations should be guided by metabolomic readouts, microbiome function, and clinically meaningful endpoints [75,90,92]. Until such evidence is available, personalized nutraceuticals should be considered a promising research and development area rather than a fully established clinical practice [24,79]. Regulatory classification, quality control, substantiation of health claims, safety monitoring, and consumer interpretation will also need to be addressed before metabotype-guided nutraceuticals can be responsibly implemented [86,89,90].

### 8.5. Challenges and Translational Priorities

Despite its potential, metabotype-guided precision nutrition faces several major challenges. First, many metabotypes have not yet been standardized. Urolithin metabotypes are relatively well characterized, while classifications for flavan-3-ol, anthocyanin, and phenolic acid metabolism remain less developed. Equol-producing status is also widely recognized, but operational definitions may vary according to the dietary challenge, biological matrix, analytical method, and cut-off used [19,21,24,25,41,42]. Therefore, standardized protocols are needed to define dietary challenge conditions, food matrix, dose, washout period, sampling time points, biological matrices, analytical thresholds, and criteria for assigning individuals to specific metabotype categories [25,27,49].

Second, the relationship between metabolite production and clinical benefit is not always clear. Producing a microbial-derived metabolite does not necessarily imply a measurable health effect, and the relevant dose, duration, exposure threshold, target population, and clinical endpoint must be defined [19,24,46,47,75]. This distinction is particularly important because metabotype classification may identify metabolic responders, but clinical translation requires evidence that these metabolic differences translate into reproducible physiological or health benefits [75,82,84,86].

Third, most available studies are short-term and involve relatively small cohorts. Larger, well-controlled, and long-term human intervention trials are needed to determine whether metabotype-guided interventions improve outcomes compared with standard dietary advice. These studies should include predefined responder analyses, standardized metabolite measurements, appropriate control groups, clinically meaningful endpoints, and replication across populations with different dietary habits, ages, health status, and microbiome backgrounds [25,28,75,82,84,86].

Fourth, regulatory and safety issues must be addressed, especially for next-generation probiotics, engineered microorganisms, and metabolite-based nutraceuticals. The introduction of metabolite-producing strains or engineered microbes into the gut ecosystem requires careful evaluation of ecological stability, horizontal gene transfer, unintended metabolic effects, colonization persistence, reversibility, and long-term safety [89,93,94,96]. In addition, health claims based on metabotype-guided interventions will require robust biomarker validation and evidence of clinical utility rather than metabolite production alone [86,90].

Finally, ethical and practical issues should be considered. Microbiome-based recommendations require biological data collection and interpretation, raising questions about privacy, accessibility, cost, data ownership, algorithmic bias, and equity. Precision nutrition should not become an approach available only to selected populations but should be developed in a way that is scientifically robust, clinically useful, interpretable, and broadly accessible [86,91,92].

### 8.6. Future Research Framework

Future research should prioritize integrated study designs that combine controlled dietary interventions, microbiome sequencing, functional metagenomics, metabolomics, clinical phenotyping, and computational modeling. A practical roadmap for advancing metabotype-guided precision nutrition could include the following steps:

1. Identification of the polyphenol precursor and expected microbial-derived metabolites;
2. Standardization of dietary challenges, including food matrix, dose, duration, and washout period;
3. Baseline assessment of gut microbiota composition, microbial gene content, and metabolic potential;
4. Controlled dietary exposure to the relevant polyphenol source;
5. Measurement of fecal, urinary, and/or circulating microbial-derived metabolites using validated analytical platforms;
6. Classification of individuals into metabotypes using explicit and reproducible criteria;
7. Evaluation of physiological and clinical responses with predefined endpoints;
8. Replication of findings across independent and geographically diverse cohorts;
9. Development and external validation of predictive models;
10. Assessment of feasibility, cost-effectiveness, regulatory requirements, and ethical implications before clinical implementation.

This framework would allow researchers to determine whether microbial metabotypes are merely descriptive biomarkers of microbial metabolism or actionable determinants of dietary responsiveness [24,25,46,47,84]. Importantly, the field should move from demonstrating that individuals differ in metabolite production toward testing whether metabotype-informed interventions improve clinically meaningful outcomes compared with conventional dietary recommendations [24,25,75,82,86]. Ultimately, the integration of microbiome science, metabolomics, controlled nutrition trials, and computational modeling may transform polyphenol research from a population-level approach into a more precise, function-based model of nutrition [1,2,26,27,28,83,84,85,86,91,92]. However, this transition will require methodological harmonization, long-term validation, and careful evaluation of clinical utility before routine implementation [25,27,28,75,86].

## 9. Conclusions

Dietary polyphenol research has progressively moved from a compound-centered view toward a host–microbiota metabolic framework. Many polyphenols show limited absorption in their native form and undergo gut microbiota-mediated transformation into more bioavailable metabolites, including urolithins, equol, enterolignans, phenyl-γ-valerolactones, and phenolic acids. This microbial conversion is highly individualized and may represent an important determinant of polyphenol bioactivity. However, the strength of evidence differs substantially among metabolite classes, biological endpoints, and study designs.

Microbial metabotypes provide a functional explanation for at least part of the interindividual variability observed in response to polyphenol-rich foods and supplements. By classifying individuals according to their capacity to generate specific microbial-derived metabolites, metabotype assessment may help distinguish metabolic responders from non-responders and improve the design of stratified nutrition studies. Nevertheless, metabotype status should not be interpreted as direct evidence of clinical benefit. Producing a microbial-derived metabolite does not necessarily imply improved physiological or health outcomes, and the relationship between metabolite exposure, tissue activity, and clinical response remains incompletely established.

The most mature examples include urolithin and equol metabotypes, whereas other proposed metabolic phenotypes, including those related to flavan-3-ol-derived valerolactones, anthocyanin-derived phenolic acids, and broader phenolic acid profiles, remain less standardized and require further validation. Clinical application of metabotype-guided nutrition is currently limited by heterogeneous dietary challenge protocols, different biological matrices, non-harmonized analytical platforms, variable cut-off definitions, limited evidence on metabotype stability, and insufficient long-term human intervention studies.

Future research should integrate microbiome profiling, functional metagenomics, metabolomics, dietary data, and clinically meaningful outcomes to clarify the relationship between microbial metabolite production and health benefits. Priority should be given to standardized challenge tests, validated biomarkers, repeated metabotype assessment, replication across diverse populations, and controlled trials testing whether metabotype-guided interventions improve outcomes compared with conventional dietary recommendations.

Overall, microbial metabotypes represent a promising mechanistic link between dietary polyphenols and individualized biological responses. However, their current value is strongest when used as functional biomarkers for research stratification and hypothesis generation rather than as established tools for routine personalized nutrition. With improved standardization, longitudinal validation, and clinically anchored trials, metabotype-based approaches may contribute to a more precise and function-based model of polyphenol nutrition.

## Figures and Tables

**Table 1:** Gut microbial pathways involved in polyphenol metabolism and metabotype evidence.

Polyphenol Class	Main Microbial Metabolites and Representative Taxa	Metabotype Relevance and Evidence Maturity	References
Ellagitannins/ellagic acid	Urolithin A, urolithin B, isourolithin A. Representative taxa include *Gordonibacter*, *Ellagibacter*, Eggerthellaceae, and *Enterocloster*.	Well-characterized urolithin metabotypes, including UM-A, UM-B, and UM-0. Evidence is relatively strong, with human dietary challenge studies and experimentally implicated bacterial producers or transformers. However, protocols and cut-offs vary across studies.	[19,20,35,36,37,38,39]
Isoflavones	Equol and *O*-desmethylangolensin. Representative taxa include *Adlercreutzia*, *Slackia*, and *Asaccharobacter*.	Recognized equol producer/non-producer phenotype. Evidence is relatively strong, with human challenge studies and mechanistic bacterial evidence. However, producer definitions differ according to matrix, analytical method, and cut-off.	[14,21,40,41,42]
Lignans	Enterodiol and enterolactone. Representative taxa include *Eggerthella*, *Blautia*, and *Lactonifactor*.	Enterolignan-producing capacity is supported by human metabolite data and microbial consortium evidence. Evidence is moderate, but standardized metabotype thresholds and clinical validation remain limited.	[22,43,44]
Flavan-3-ols	Phenyl-γ-valerolactones and phenylvaleric acids. Representative taxa include *Flavonifractor*, *Bacteroides*, and Lachnospiraceae.	High interindividual variability has been reported, but formal metabotype classification remains emerging. Many taxa are associated or inferred contributors rather than fully validated producers.	[11,12,13,17,24,32]
Anthocyanins	Protocatechuic acid, vanillic acid, and related phenolic acids. Representative taxa include *Bifidobacterium*, *Lactobacillus*, and *Bacteroides*.	Variable phenolic acid profiles have been described, but no standardized metabotype classification exists. Evidence is mainly in vitro, animal, or associative human metabolomics.	[12,17,18,31,35]
Flavonols	Phenolic acid derivatives. Representative taxa include *Bacteroides*, *Eubacterium*, and *Flavonifractor*.	Interindividual variability in metabolite production is recognized, but evidence remains emerging and strain-dependent. Current data are insufficient for a validated formal metabotype.	[11,12,17,32]
Phenolic acids	Ferulic acid derivatives, benzoic acid derivatives, and related metabolites. Representative taxa include *Bacteroides*, *Lactobacillus*, and *Bifidobacterium*.	These metabolites contribute to the circulating phenolic metabolite pool, but evidence is mostly indirect and influenced by diet, host metabolism, and microbial transformation. Not yet suitable for standardized metabotype classification.	[11,17,31,32]

Abbreviations: UM-A, urolithin metabotype A; UM-B, urolithin metabotype B; UM-0, non-urolithin producer. Note. Evidence maturity refers to the degree to which a polyphenol-related metabolic phenotype has been reproduced in humans and linked to experimentally supported microbial pathways. Listed taxa include experimentally implicated producers or transformers as well as taxa associated with metabolite production; therefore, taxa should not always be interpreted as confirmed causal producers in humans.

**Table 2:** Biological activities of microbial-derived polyphenol metabolites and the level of supporting evidence.

Metabolite Class	Main Biological Relevance	Evidence Maturity and Limitations	References
Urolithins, especially urolithin A	Mitochondrial quality control, mitophagy, muscle function, cellular resilience, and possible endothelial or metabolic effects.	Most mature example. Human supplementation studies support effects on selected mitochondrial and muscle-related biomarkers, but evidence mostly concerns direct urolithin A supplementation rather than endogenous production after ellagitannin-rich foods. Long-term clinical relevance remains insufficiently established.	[19,37,65,66,67,68,72]
Equol	Estrogen receptor-mediated signaling, cardiometabolic regulation, vascular function, and endocrine-related effects.	Moderate evidence. Equol-producer status is recognized, but definitions, challenge protocols, biological matrices, and cut-offs differ across studies. Clinical relevance varies by dose, population, and endpoint.	[14,21,75,82]
Enterolignans, including enterodiol and enterolactone	Hormone-related pathways, metabolic regulation, cardiometabolic markers, and inflammatory responses.	Moderate but less standardized than urolithin and equol phenotypes. Human metabolite data are available, but standardized metabotype thresholds and clinical outcome validation remain limited.	[22,43,44,75]
Phenyl-γ-valerolactones and phenylvaleric acids	Endothelial function, nitric oxide signaling, redox-sensitive pathways, mitochondrial signaling, and metabolic regulation.	Emerging evidence. Circulating metabolites are documented after flavan-3-ol intake, but causal links with vascular or metabolic outcomes remain incompletely established. Formal metabotype classifications are still limited.	[13,17,24,72,75]
Phenolic acids from anthocyanins and other flavonoids	Redox modulation, cellular stress responses, endothelial signaling, neuronal viability, and metabolic pathways.	Limited to emerging evidence. Data are mainly from in vitro, animal, and associative human metabolomics studies. Attribution to specific microbial pathways is difficult, and no widely accepted metabotype classification exists.	[17,31,35,75]
Mixed microbial phenolic profiles	Systems-level modulation of inflammation, oxidative stress, gut barrier function, gut–brain signaling, and metabolic homeostasis.	Emerging evidence. Relevant for multi-omics precision nutrition models, complex metabolite mixtures make causal attribution difficult. Human validation is endpoint-specific and heterogeneous.	[24,83,84,85]

Note: Evidence strength refers to the overall maturity of the evidence linking metabolite production to biological or clinical effects. “Relatively strong” indicates repeated human supplementation or intervention evidence supported by mechanistic studies; “moderate” indicates human metabolite or producer-status data with incomplete clinical validation; “emerging” indicates mechanistic, experimental, or associative evidence requiring further controlled human validation.

## Data Availability

No new data were created or analyzed in this study. Data sharing is not applicable to this article.

## Abbreviations

The following abbreviations are used in this manuscript:

AI	Artificial intelligence
ROS	Reactive oxygen species
UM	Urolithin metabotype
UM-A	Urolithin metabotype A
UM-B	Urolithin metabotype B
UM-0	Non-urolithin producer
